# Development and comparative study of a *pat/bar* real‐time PCR assay for integrating the screening strategy of a GMO testing laboratory

**DOI:** 10.1002/jsfa.10235

**Published:** 2020-01-17

**Authors:** Daniela Verginelli, Annalisa Paternò, Maria Laura De Marchis, Cinzia Quarchioni, Daniela Vinciguerra, Pamela Bonini, Stefania Peddis, Cristiana Fusco, Marisa Misto, Cristina Marfoglia, Francesco Pomilio, Ugo Marchesi

**Affiliations:** ^1^ National Reference Laboratory for GM Food and Feed, GMO Unit Istituto Zooprofilattico Sperimentale del Lazio e della Toscana “Mariano Aleandri” Rome Italy; ^2^ Igiene delle tecnologie alimentari e dellʼalimentazione animale Istituto Zooprofilattico Sperimentale dellʼAbruzzo e Molise "G. Caporale" Teramo Italy

**Keywords:** GMO, multiplex real‐time PCR, screening, pre‐spotted plates, *pat* gene, *bar* gene

## Abstract

**BACKGROUND:**

The number and variety of genetically modified organisms (GMOs) used globally for the production of food and feed, and potentially circulating in the European Union (EU), is constantly increasing. This implies an additional effort for the EU enforcement laboratories to optimize available resources, to contain costs and time. A well established approach for streamlining the analytical workflow is the introduction of a screening step, typically based on a smart set of real‐time polymerase chain reaction (PCR) screening methods. The multiplexing strategy, allowing the detection of several screening elements simultaneously, is a further optimization of this step.

**RESULTS:**

In this study, we present the validation of a real‐time PCR duplex assay for the *pat* and *bar* screening elements to be easily incorporated in the GMO diagnostic routine. We also provide a comparison between this method and the related singleplex and pre‐spotted assays.

**CONCLUSION:**

Our results fully respect all the validation parameters suggested by the Minimum Performance Criteria of the European Network of GMO Laboratories. Furthermore, the duplex assay is equivalent in terms of performance compared to the other two methods, but it shows a higher overall flexibility and cost effectiveness. © 2019 The Authors. *Journal of The Science of Food and Agriculture* published by John Wiley & Sons Ltd on behalf of Society of Chemical Industry.

## INTRODUCTION

According to the EU legislation regulating the presence of genetically modified organisms (GMOs) in food and feed, the enforcement laboratories must verify compliance with the requirements of labeling and traceability for all the authorized genetically modified (GM) events.^1^, ^2^ There are currently 125 authorized GM events in the EU, 24 GM events with a pending authorization procedure, seven GM events with an expired authorization and nine events subject to Commission Decisions on withdrawal from the market (comprising stacked events).^3^ The growing number of GM authorizations in the EU reflects the constantly increasing share of GM events circulating worldwide.^4^ The identification of each event is possible by using event‐specific real‐time polymerase chain reaction (PCR) methods but sometimes this approach takes time and incurs costs, due to the large amount of GMOs.

An efficient and consolidated analytical strategy is the screening‐based approach: a panel of screening elements recurring in transgenic constructs can be selected based on potentially GM plant species detected in the sample and analyzed before using event‐specific methods.^5^ Many different screening methods have already been developed and validated in singleplex real‐time PCRs.^6^, ^7^, ^8^, ^9^ Among these, a panel consisting of seven screening elements has been adopted by the National Reference Laboratory for GM food and feed in Italy: the cauliflower mosaic virus 35S promoter (*p35S*), the nopaline synthase terminator (*tNOS*), the phosphinothricin *N*‐acetyl transferase gene from the bacterium *Streptomyces hygroscopicus* (*bar*) and from *Streptomyces viridochromogenes* (*pat*), the neomycin phosphotransferase II (*nptII*), the 5‐enolpyruvylshikimate−3‐phosphate synthase gene (*CP4 epsps*) and the junction between the chloroplast transit peptide and the CP4 epsps gene (*ctp2‐CP4 epsps*).^10^ To simplify these qualitative steps and favor a ‘multi‐target’ approach, ‘pre‐spotted’ plates (PSPs) with ready‐to‐use reagents have been developed. Pre‐spotted plates allow the simultaneous detection of different taxa, screening and event‐specific targets in a pre‐defined or customizable setting, by a combination of real‐time singleplex assays in the same plate.^11^

A further solution to the optimization of the available resources is the development of a multiplex real‐time PCR method combining two or more targets in one reaction. Moreover, less variable results for the co‐amplification of different targets can be achieved. In recent years, several authors presented methods based on detection and identification of GMO targets using multiplex real‐time PCR.^12^, ^13^, ^14^, ^15^, ^16^, ^17^, ^18^, ^19^ One of the most critical aspects in the development of multiplex PCR methods is the optimization of the experimental conditions with particular regard to the balancing of the components of the reaction mixture. For this reason almost all of the assays cited above, with the only exception of the p35S/tNOS duplex system described by Waiblinger *et al*.,^12^ were based on the use of master mixes optimized for multiplexing. Although very precious in the optimization phase, these reagents are generally more expensive than the conventional ones and often impose specific cycling conditions that may require separate work sessions. As a consequence, it may not be convenient to integrate these methods directly into the diagnostic routine, especially if the laboratory already has several other tests in use to be performed in parallel. To overcome these limitations, we sought to develop a duplex assay, compatible with the reagents and thermal cycling conditions of singleplex assays, focusing on *pat* and *bar* genes. Both these genes confer glufosinate tolerance to the recipient plant and are among the most widely used elements to produce GM events. Actually, 40 GM events with non stacked traits expressing the *pat* gene and 31 GM events with non stacked traits expressing the *bar* gene, are known.^20^ In our laboratory, we adopted the EU reference methods QL‐ELE‐00‐014 and QT‐ELE‐00‐002 for *bar* and *pat* single screening assays, respectively.^7^, ^8^ Both methods are also available in the PSPs mentioned above, developed by the European Union Reference Laboratory for GM Food and Feed (EURL GM‐FF).^11^

In this study, we present our in‐house validation of a *pat*/*bar* duplex real‐time PCR method, according to the document ‘Definition of Minimum Performance Requirements for Analytical Methods of GMO Testing’ (MPR) and considering other guidelines for the validation of qualitative methods.^21^, ^22^ We also provide a comparison with the respective singleplex methods and the pre‐spotted screening assays.

## MATERIALS AND METHODS

### Reference materials and DNA samples

Certified reference materials (CRMs) were purchased from the Institute for Reference Materials and Measurements (IRMM, Geel, Belgium) and the American Oil Chemistsʼ Society (AOCS, Urbana, IL, USA). Real‐life samples, selected by the Italian National Reference Laboratory for GM food and feed, were used for evaluating the validation parameters.

### DNA extraction

The extraction of DNA from all CRMs and samples was performed using ION‐Force DNA extractor FAST (Generon S.P.A. Italy) according to the manufacturerʼs instructions starting from 200 mg of CRMs and 2,5 or 5 g of routine samples. Concentration and purity of extracted DNA were evaluated using a biophotometer (Eppendorf, Hamburg, Germany).

### Primers and probes

The primers and probes for this study were supplied by Eurofins Genomics (Ebersberg, Germany). The sequences are listed in Table 1. The event‐specific singleplex real‐time PCR assays were performed according to the reference methods of the EURL GM‐FF.^23^

**Table 1:** Primer and probe sequences adopted for the *pat* and *bar* singleplex and duplex real‐time PCR assays

Target	Name	Sequence (5′ − 3′)	Amplicon size (bp)	References
*bar*	RapB‐F1	ACAAGCACGGTCAACTTCC	60	Grohmann *et al*., 2009^8^
	RapB‐R1	GAGGTCGTCCGTCCACTC		
	RapB‐S1	**FAM**‐TACCGAGCCGCAGGAACC‐**TAMRA** b		
*pat*	KVM‐5	TTGAGGGTGTTGTGGCTGGTA	68	Weighardt *et al*., 2004^7^
	KVM‐6	TGTCCAATCGTAAGCGTTCCT		
	TaqMan Pat1	a **FAM**‐CTTCCAGGGCCCAGCGTAAGCA‐**TAMRA** b		

a For the duplex assay the FAM reporter of the *pat* probe was replaced with the Yakima Yellow (YY) reporter.

b For the duplex assay the TAMRA quenchers were replaced with BBQ and BHQ1 for the *bar* and *pat* probe, respectively.

### Real‐time PCR

Real‐time PCR reactions were performed on a 7900HT Real‐Time PCR System (Life Technologies, Foster City, CA, USA). A ready‐to‐use TaqMan Universal Master Mix (2x) (Thermo Fisher Scientific, Waltham, MA, USA) at 1 × final concentration was used. The amplification was performed in a final volume of 25 μL with a DNA amount of 100 ng for each target. The final concentration of primers and probes in the singleplex pat assay was 0.4 μM and 0.2 μM, respectively, while in the singleplex bar assay final concentration of the primers and probes was 0.14 μM and 0.1 μM, respectively. In the duplex reaction the final concentrations were 0.4 μM for primers and 0.2 μM for probes for both systems. The following cycling parameters were used: UNG (Uracil N‐glycosylase) pre‐treatment of 2 min at 50 °C, initial activation of the polymerase for 10 min at 95 °C followed by 45 cycles of 95 °C for 15 s and 60 °C for 60 s. ‘Pre‐spotted’ plate assays contained 0.6 μM of each primer and 0.2 μM of the FAM (6‐Carboxyfluorescein)/TAMRA (5‐Carboxytetramethylrhodamine)‐labeled probes.

The SYBR Green chemistry was adopted using Power SYBR Green PCR Master Mix (Applied Biosystems). The reaction was performed in a final volume of 20 μL with a DNA amount of 100 ng. The final concentration of primers was 0.4 μM. The PCR conditions were as follows: 10 min at 95 °C, 40 cycles of 15 s at 95 °C and 1 min at 60 °C ± 1.75 °C/min from 60 °C to 95 °C.

### Droplet digital PCR assays

Droplet digital PCR (ddPCR) assays were performed in a total reaction volume of 20 μL containing ddPCR Supermix for Probes (Bio‐Rad, Pleasanton, CA), 4 μL of DNA template, 0.6 μM primers and 0.25 μM probes. Data generated by the QX100 droplet reader were analyzed by Quantasoft 1.7.4.0917 software rejecting PCR reactions (single wells) with a number of droplets that was too low (<10 000).

### Statistical analysis

Data are presented as the average value ± standard deviation. Cq (cycle quantification value: PCR cycle number at which the reaction curve intersects the threshold line) data were analyzed by Studentʼs paired *t*‐test. Results were considered statistically significant only with *P* values lower than 0.05.

## RESULTS

### Specificity

To verify the unintended generation of additional amplicons due to the interaction of forward and reverse primers of each system, a melting curve analysis was performed testing BT11 1% and T304 1% positive controls, both individually and in a mixture, using SYBR Green chemistry. No additional peaks were observed (Fig. S1). The absence of additional amplicons was also verified by visualizing the duplex PCR products on an agarose gel. Only bands of the expected length were observed (Fig. S2). We tested 22 target and 38 non‐target CRMs by comparing the expected and verified results for each target. Specificity was checked experimentally by measuring the false positive rate (type I error rate) and the false negative rate (type II error rate) with an acceptance criterion of *α* and *β* ≤5%. No false positive and false negative results were observed (Table 2).

**Table 2:** Specificity test for the *pat/bar* duplex real‐time PCR assay

Species	GM event	Reference material	*bar*		*pat*	
			Expected	Verified	Expected	Verified
Cotton	MON1445	AOCS 0804‐B	A	−	A	−
	MON15985	AOCS 0804‐D	A	−	A	−
	MON513	AOCS 0804‐C	A	−	A	−
	LL25	AOCS 0306‐E	P	+	A	−
	GHB614	AOCS 1108‐A	A	−	A	−
	3006‐210‐23 × 281‐24‐236	ERM‐BF422‐b	A	−	P	+
	MON88913	AOCS 0906‐D	A	−	A	−
	T304‐40	ERM‐BF429‐c	P	+	A	−
	GHB119	ERM‐BF428‐c	P	+	A	−
Maize	Bt11	ERM‐BF412‐f	A	−	P	+
	DAS1507	ERM‐BF418‐d	A	−	P	+
	DAS59122	ERM‐BF424‐c	A	−	P	+
	GA21	AOCS 0407‐B	A	−a	A	−
	MIR162	AOCS 1208‐A	A	−	A	−
	MIR604	ERM‐BF423‐d	A	−	A	−
	MON810	ERM‐BF413‐gk	A	−	A	−
	MON88017	AOCS 0406‐D	A	−	A	−
	NK603	ERM‐BF415‐d	A	−	A	−
	T25	AOCS 0306‐H	A	−	P	+
	MON89034	AOCS 0906‐E	A	−	A	−
	MON87460	AOCS 0709‐A	A	−	A	−
	MON87427	AOCS 0512‐A	A	−	A	−
	3272	ERM‐BF420‐b	A	−	A	−
	DAS40278‐9	ERM‐BF433‐d	A	−	A	−a
	5307	AOCS 0411‐D	A	−	A	−
	MON863	ERM‐BF416‐d	A	−	A	−a
	Bt176	ERM‐BF411‐d	P	+	A	−
	98140	ERM‐BF427‐d	A	−	A	−
	LY038	pENGL‐00‐01/06‐01^b^	A	−	A	−
	DAS59132‐8	1% DAS‐59132‐8 (E‐32)^c^	A	−	A	−a
Flax	FP967	pENGL‐00‐EM01/09‐01^b^	A	−	A	−
Oilseed rape	MS8	AOCS 0306‐F	P	+	A	−
	RF3	AOCS 0306‐G	P	+	A	−
	T45	AOCS 0208‐A	A	−	P	+
	GT73 (RT73)	AOCS 0304‐B	A	−	A	−
	MON88302	AOCS 0911‐A	A	−a	A	−
	DP‐73496‐4	ERM‐BF434‐E	A	−	A	−
	MS1	AOCS 0711‐A	P	+	A	−
	RF1	AOCS 0711‐B	P	+	A	−
	RF2	AOCS 0711‐C	P	+	A	−
	Topas19/2	AOCS 0711‐D	A	−	P	+
Soybean	MON40‐3‐2	ERM‐BF410‐d	A	−	A	−
	A2704‐12	AOCS 0707‐B	A	−	P	+
	MON89788	AOCS 0906‐B	A	−	A	−
	DP‐356043‐5	ERM‐BF425c	A	−	A	−
	A5547‐127	AOCS 0707‐C	A	−	P	+
	MON87701	AOCS 0809‐A	A	−	A	−
	DP‐305423‐1	ERM‐BF426‐c	A	−	A	−
	CV‐127‐9	AOCS 0911‐C	A	−	A	−
	MON87705	AOCS 0210‐A	A	−	A	−
	MON87708	AOCS 311‐A	A	−	A	−
	MON87769	AOCS 0809‐B	A	−	A	−
	FG72	AOCS 0610‐A	A	−	A	−
	DAS‐81419‐2	ERM‐BF437‐e	A	−	P	+
	DAS‐68416‐4	ERM‐BF432‐c	A	−	P	+
	DAS‐44406‐6	ERM‐BF436‐d	A	−	P	+
Potato	EH92‐527‐1	ERM‐BF421‐b	A	−	A	−
Sugar beet	H7‐1	AOCS 1206‐B	A	−	A	−
Rice	LL62	AOCS 0306‐I	P	+	A	−
	LL601	1% LLRICE601^c^	P	+	A	−
	BT63	pENGL‐00‐EM02/06‐01M^b^	A	−	A	−
	KEFENG6	5% KeFeng6^c^	A	−	A	−
Papaya	55‐1	Bayerisches LGL DNA^d^	A	−	A	−

A, absence; P, presence; +, element detected (Cq values <37); *−*, element not detected.

a The presence of the element was detected with Cq values ≥37 due to GM contamination (data not shown). These reference materials were considered negative.

b Plasmid DNA supplied by the European Union Reference Laboratory for GM Food and Feed (EU‐RL GMFF).

c Genomic DNA supplied by the European Union Reference Laboratory for GM Food and Feed (EU‐RL GMFF).

d Genomic DNA supplied by Bavarian Health and Food Safety Authority (LGL) Germany.

### PCR efficiency and linearity

A mixture of DNA containing an equal number of genomic copies of RF1 100% GM rapeseed and A5547 100% GM soybean, was prepared to test the efficiency and linearity of the *pat*/*bar* duplex system.

The starting copy number of haploid genome equivalents (HGE) was quantified by using the ddPCR. To obtain a sufficient amount for each target, two 100% GM events were selected: RF1 rapeseed and A5547 soybean for *bar* and *pat*, respectively. The copy number of both targets was quantified by evaluating two ddPCR replicates. The average value obtained was used to calculate the starting concentration (Fig. S3). For each system, five serial dilutions containing 5120, 1280, 320, 80, and 20 HGE were obtained.

Four plates including six replicates of each dilution point were run under repeatability conditions resulting in 24 data / dilution points. The average Cq of each point was plotted against the log_10_ HGE and a linear regression analysis was performed (Table S1). The average slopes of the regression lines were −3.34 and −3.42, corresponding to an efficiency of 99.3% and 96.3% for the *bar* and *pat* system. In both cases, the average R^2^ was 1.00 (Table 3). All these values met the acceptability criteria suggested by Broeders *et al*. concerning methods intended for qualitative use.^22^

**Table 3:** Linearity and efficiency assessment for the *pat/bar* duplex real time PCR assay

	*bar*			*pat*		
	Slope	R^2^	Efficiency (%)	Slope	R^2^	Efficiency (%)
Run 1	−3.24	1.000	103.4	−3.42	0.999	96.1
Run 2	−3.36	0.999	98.5	−3.39	1.000	97.4
Run 3	−3.43	0.997	95.8	−3.41	1.000	96.3
Run 4	−3.34	1.000	99.4	−3.44	1.000	95.2
Average	−3.34	0.999	99.3	−3.42	1.000	96.3

### Asymmetric limit of detection (LOD 20_asym_)

As described in the MPR document, a multiplex assay, the asymmetric LOD (LOD_asym_) needs to be determined for each assay, to evaluate if they both perform equally and if competition between the targets occurs.^21^

The RF1 100% rapeseed and A5547 100% soybean CRM were selected as positive CRMs for *bar* and *pat*, respectively. Based on the previous ddPCR quantification, for both systems, one target was set at 20 HGE, whereas the other one was set at 20 000, 10 000 and 5000 HGE, resulting in a 1/1000, 1/500 and 1/250 ratio. Each point was run in six replicates per target. A positive signal (Cq < 40) was detected for all the six replicates for 20 copies of target in a background of 20 000 copies of non‐target.

### Robustness

To evaluate the robustness of the system, three final reaction volumes (24, 25, and 26 μL), two master mixes (2x TaqMan Universal Master Mix from Thermo Fisher Scientific and 2x QuantiTect Multiplex PCR NoROX Mastermix from Qiagen) and two PCR instruments (7900HT and QuantStudio 7 from Thermo Fisher Scientific) were tested on oilseed rape T45 100% and RF1 100% MDRs. The assay was performed using a mix containing 20 HGE of each target in three replicates, as suggested by Broeders *et al*.^22^ All the replicates gave positive results (Table S2).

### Method transferability

In order to test method transferability, 20 blind positive / negative DNA samples (CRMs), with the same GM content tested for our in‐house validation, were sent to the Istituto Zooprofilattico dellʼAbruzzo e del Molise. All the results were in agreement with the expected pattern of presence / absence for *pat* and *bar* (Table S3).

### Comparison between duplex PCR, singleplex PCR and EURL PSP assays on real life samples

The duplex *pat*/*bar* real‐time PCR assay was compared to its singleplex and PSPs counterparts, testing real life samples (different matrices of feed and processed food). Each sample was extracted twice and three replicates per extraction point were tested by real‐time PCR. Table 4 reports the resulting average Cq values related to *pat* and *bar* screening elements. We considered the sample negative for the presence of the element under investigation (Undet) if each extraction replicate gave at least an ‘undetermined’ signal or a Cq > 40. In order to avoid any source of variability, all the master mixes were prepared shortly before use. Furthermore, all real‐time PCR runs were performed with the same batch of mastermixes. Given the good quality of the amplification curves (data not shown), we collected all the Cq values by setting the threshold to the default value of *Δ*Rn equal to 0.2, to compare the performance of the three assays. Genetically modified events were indicated according to the results of routine analyses. Almost in all cases, the detected events could explain the observed pattern of positivity to *pat* and *bar*. The unexpected positivity for *pat* and *bar* element in sample n.1 and n.6, respectively, was probably due to the presence of a contaminating unidentified GM event. The three assays always gave concordant results with the only exception of sample n.6, for which bar was detected in all the six replicates only by PSPs. Two samples were negative for the presence of both *pat* and *bar* elements, one sample was positive for the presence of *bar*, and nine samples were positive for the presence of *pat*. In two samples, both *pat* and *bar* were detected.

**Table 4:** Comparison between duplex, singleplex and pre‐spotted assays performance on GMO‐positive real‐life samples

Sample ID	Detected GM Events	Real life samples	Label ingredients	*bar*			*pat*		
				Duplex	Singleplex	PSP	Duplex	Singleplex	PSP
1	BT176	Maize grain	maize	36.98 ± 0.79*	37.68 ± 0.62**	36.04 ± 0.57***	36.90 ± 0.62	36.58 ± 1.41	36.47 ± 0.64
2	LL601	Ready‐cooked rice	rice	35.49 ± 0.52*	37.01 ± 0.32**	34.78 ± 0.30***	Undet	Undet	Undet
3	DAS1507 T25	Pet food	rice, maize	Undet	Undet	Undet	36.64 ± 0.66	37.17 ± 0.65	36.65 ± 0.58
4	DAS1507 T25	Pet food	rice, maize	Undet	Undet	Undet	38.08 ± 0.24	37.49 ± 0.75**	36.47 ± 0.59***
5	BT11 DAS1507	Animal feed	maize, soybean	Undet	Undet	Undet	31.59 ± 0.18*	30.93 ± 0.09**	30.65 ± 0.12***
6	—	Animal feed	maize, soybean, rice, oilseed rape, sugarbeet	Undet	Undet	38.15 ± 0.61	Undet	Undet	Undet
7	—	Animal feed	declared GM maize and soybean	Undet	Undet	Undet	Undet	Undet	Undet
8	A2704, RF3, MS8	Animal feed	maize, soybean, sugarbeet, rapeseed	36.19 ± 0.49*	35.79 ± 0.41	35.93 ± 0.69	35.13 ± 0.44*	36.58 ± 0.40	36.7 ± 0.68
9	—	Animal feed	declared GM maize and soybean	Undet	Undet	Undet	Undet	Undet	Undet
10	BT11 DAS1507	Maize grain	maize	Undet	Undet	Undet	35.04 ± 0.56*	34.02 ± 0.43	33.77 ± 0.17***
11	BT11 DAS1507	Animal feed	maize	Undet	Undet	Undet	35.64 ± 0.27*	34.36 ± 0.32	34.40 ± 0.31***
12	BT11 DAS1507	Animal feed	maize	Undet	Undet	Undet	26.54 ± 0.09*	26.10 ± 0.18**	25.80 ± 0.06***
13	BT11 DAS1507	Animal feed	maize	Undet	Undet	Undet	30.91 ± 0.60*	29.92 ± 0.63**	29.68 ± 0.53***
14	BT11 DAS1507	Animal feed	maize	Undet	Undet	Undet	29.47 ± 0.38*	28.55 ± 0.54	28.23 ± 0.23***
15	BT11 DAS1507	Animal feed	maize, soybean	Undet	Undet	Undet	30.10 ± 0.11*	29.75 ± 0.26	29.33 ± 0.26***

Resulting average values (± SD) of Cq starting from six replicates of real‐time PCR are reported.

*P < 0.05 in the comparison between the duplex and singleplex assay.

**P < 0.05 in the comparison between the singleplex and PSP assay.

***P < 0.05 in the comparison between the duplex and PSP assay.

According to a general trend, the average Cq value of PSP *bar* and *pat* assays were lower than singleplex and duplex methods, with a statistically significant difference for two out of four *bar* positive samples and four out of eleven *pat* positive samples (*P* < 0.05).

### Applicability of the integrated screening strategy

To demonstrate the integrability of the novel *pat/bar* duplex assay in the pre‐existing screening strategy, 2 CRMs containing *pat* (BT11 and T45), 2 CRMs containing *bar* (BT176 and RF1), two mixtures obtained by mixing CRMs containing both targets, and three real‐life samples positive for the presence of *pat*, *bar* or both were tested in the same run with a panel composed by the novel duplex method in combination with the other five qPCR methods already in use at the Italian NRL (*P35S, TNOS, NPTII, CP4 EPSPS, CTP2‐CP4 EPSPS*). As shown in Table 5, the results obtained perfectly reflect the expected values for each material analyzed.

**Table 5:** Applicability of the integrated screening strategy

Sample ID	Real life samples and CRMs	P35S		TNOS		NPTII		BAR		PAT		CP4‐EPSPS		CTP‐CP4EPSPS	
		Expected	Verified	Expected	Verified	Expected	Verified	Expected	Verified	Expected	Verified	Expected	Verified	Expected	Verified
1	Maize grain	P	+	A	−	A	−	P	+	P	+	A	−	A	−
2	Animal feed	P	+	P	+	A	−	A	−	A	−	P	+	P	+
3	Animal feed	P	+	P	+	A	−	A	−	P	+	P	+	P	+
4	Maize BT11 5% + maize BT176 5%	P	+	P	+	A	−	A	+	P	+	A	−	A	−
5	Oilseed rape T45 100% + oilseed rape RF1 100%	P	+	P	+	P	+	P	+	P	+	A	−	A	−
6	Maize BT11 5%	P	+	P	+	A	−	A	−	P	+	A	−	A	−
7	Oilseed rape T45 100%	P	+	A	−	A	−	A	−	P	+	A	−	A	−
8	Maize BT176 5%	P	+	A	−	A	−	P	+	A	−	A	−	A	−
9	Oilseed rape RF1 100%	A	−	P	+	P	+	P	+	A	−	A	−	A	−

Single or combined CRMs and real‐life samples positive for the presence of *pat*, *bar*, or both were tested in the same run (‘verified’ column with +: element detected (Cq values <37) ‘verified’ column with −: element not detected or Cq values >37). The results are compared with the expected values (A: Absence. P: Presence) on the basis of either the certified content (samples 4 to 9) or previous routine analyses (samples 1,2 and 3).

## DISCUSSION

Developing a multiplex PCR for screening methods is a good way to reduce the overall costs and turnaround times of GMO analyses. Particularly, Cottenet *et al*. in 2013 developed a ‘ready‐to‐use’ 384‐well PSP grouping 47 targets (taxon, screening and event‐specific) in 23 real‐time triplex PCR systems, providing an internal control of amplification in each well.^14^ Two duplex real‐time PCR methods targeting *p35S*/*tNOS* and *bar*/*pat*, respectively, and a triplex real‐time PCR targeting *p35S*/*tNOS*/*CP4 epsps* have been validated and published.^12^, ^15^, ^18^ The *p35S* and *tNOS* were combined with the Figworth Mosaic Virus promoter (*pFMV*) and the Cauliflower Mosaic Virus 35S terminator (*t35S*) in a tetraplex real‐time PCR system.^16^ Furthermore, Köppel *et al*. in 2014 developed two real‐time PCR multiplex assays. The first one was a pentaplex system specific for the screening elements *p35S* and *tNOS*, the taxon‐specific genes for maize (Major high‐mobility group protein) and soybean (Lectin) and the donor organism Cauliflower mosaic virus. The latter was a tetraplex system targeting four screening elements: the *pFMV*, *bar*, and *pat* genes and the *ctp2‐CP4 epsps* construct.^17^ In all these cases, a Taqman chemistry was adopted, allowing the use of probes labeled with different fluorophores, each associated to a specific target. Generally, the most critical aspect of setting up a multiplex assay is linked to the crosstalk among fluorescence signals, so that a maximum of five or six fluorophores can be combined. Bahrdt *et al*. in 2010 developed a hexaplex assay, comprising the screening methods for *p35S*/tNOS/pFMV, two modification specific‐assays for LY (junction between *ctp* and *cordapA* gene) and SAMS (junction between *S*‐adenosyl‐l‐methionine and the *gm‐hra* gene) and an internal positive control system for the detection of PCR inhibitors.^13^ Recently, a new strategy of multiplexing real‐time PCR has been developed, based on the simultaneous detection of six GMO targets exploiting the signal emitted by a single fluorophore (FAM) in a FAST mode. This kind of approach could be very useful, especially to quickly screen samples negative for all the selected targets.^19^

With the only exception of the *p35S*/*tNOS* duplex system, published by Waiblinger *et al*., all the above mentioned real‐time PCR multiplex assays have been developed with the use of master mixes optimized for multiplexing. These reagents are more expensive than those conventionally adopted for singleplex PCR assays and often require a specific thermal profile (TaqMan Environmental Master Mix 2.0, AmpliTaq Gold DNA Polymerase from Thermo Fisher Scientific, Taqman FAST Advanced Master Mix, QuantiFast Multiplex PCR Kit and QuantiTect Multiplex PCR Kit from QIAGEN). The introduction of these assays into the routine diagnostic workflow could therefore require the establishment of sessions dedicated to a few groups of targets, which might be inconvenient in the end.

One of the aims of this study was to develop and validate a cost‐ and time‐efficient duplex PCR assay for *pat/bar*, with the same analytical conditions as the routine methods, already adopted by the laboratory for the same analytical step.

Huber *et al*. developed a *pat*/*bar* duplex real‐time PCR with the same screening method for *bar*, and the QL‐ELE‐00‐025 method for *pat*.^15^, ^18^, ^24^ According to the JRC ‘GMO‐MATRIX’ module, the two *pat* methods share the same analytical coverage.^25^ Furthermore, both the *pat*/*bar* duplex methods meet all the validation criteria suggested by the MPR. However, the assay developed by Debode *et al*. is based on the use of a real‐time PCR mastermix, optimized for multiplexing with a specific thermal profile, requiring a dedicated session. Moreover, based on the current available price list, the use of such master mix requires about 0.50 euros more per well than the mix adopted for our method. Huber *et al*. used FAM (emission peak at 520 nm) and HEX (emission peak at 556 nm) to label the *bar* and *pat* assay probe, respectively. In our case, the use of the TaqMan Universal Master Mix posed the problem of the presence of ROX as passive reference dye (emission peak at 602 nm), which showed a marked signal overlap with the HEX fluorochrome (data not shown). We produced better discrimination of the emitted signals by marking the probe of the *pat* assay with the Yakima Yellow fluorochrome (emission peak at 549 nm).

Looking at the comparison between the *pat*/*bar* duplex assay, *pat* and *bar* singleplex assays, and PSPs, the three methods showed a substantial equivalence in terms of screening performance. The most significant trend was related to PSPs, often being associated with lower average Cq values. In one case, PSPs showed a sensitivity higher than the other two assays, detecting the presence of the *bar* gene. This overall effect was probably due to the higher concentration of primers spotted in PSPs. The ‘ready‐to‐use’ setting reduces the risk of contamination and error by the operator when preparing the reaction mix. In addition, PSPs provide fresher reagents because they are used immediately after being reconstituted. However, PSPs are a less flexible system, because the pre‐set pattern does not always allow the simultaneous optimization of turnaround times and costs, especially when few samples are to be analyzed or in laboratories with a limited activity. Furthermore, depending on the complexity in the composition of the plate, the PSPs price may vary from a minimum of 0.70 euros per well up to a maximum of 1.50 euros per well, in addition to the master mix (Gatto F, 2019, pers. comm.).

In summary, what we describe is the validation of an innovative element to be embedded, in a compatible way, in the overall analytical strategy of an experienced GMO testing laboratory. The work's added value is related to the comparison among three different screening approaches. Given the comparability of the results, a decision‐making system could be considered, based on a case‐by‐case assessment of the screening method to use. For example, the adoption of the multiplex or PSPs could be more convenient than singleplex in the case of mixed matrices with more than one ingredient (which most probably could contain both elements). The adoption of PSPs could be advantageous in periods of intensive workload. Other aspects to be considered when choosing the best screening strategy could be related to the plant species composing the samples and the analytical request. For example, in the case of monitoring plans focusing on maize‐based products, *pat* is definitely more informative than *bar*, as this last element is reported only in a limited number of GM maize events not authorized in the EU. So a singleplex *pat* assay could be sufficient for screening authorized maize events. In conclusion, itʼs not possible to define a universal screening strategy for GMO testing. The three approaches are all eligible, depending on the specific need and situation (number and complexity of the samples and type of analytical request). The important aspect is to make them easily interchangeable within the laboratory by harmonizing the experimental protocols.

## CONCLUSIONS

The duplex *pat/bar* assay, developed and validated in this study, represents an alternative method that can be perfectly integrated into the standard sessions of GMO diagnostic routines, favoring an optimization of resources, costs, and response time. In conclusion, the use of the same mixture and cycling conditions implies a simpler organization of the work and could be recommended as a general technical solutions for optimizing other multiplex methods.

## AUTHORSHIP

D.Ve. conceived the experimental design, performed the acquisition and interpretation of data, and revised the manuscript. A.P. and M.L.D.M. performed the interpretation of data, drafting, and revision of the manuscript. C.Q., D.Vi., P.B., S.P., C.F. and M.M. carried out the experimental work and acquisition of data. C.M. and F.P. supported the experimental work and acquisition of data (please refer “method transferability”); U.M. supervised the project, revised the manuscript, and approved the final version.

## Supporting information


**Appendix S1.** Supporting Information
