# Supplementary material for: Development and comparative study of a pat/bar real‐time PCR assay for integrating the screening strategy of a GMO testing laboratory
Source: J Sci Food Agric. 2020 Jan 17;100(5):2121–9. doi: 10.1002/jsfa.10235 (PMC7384061; doi:10.1002/jsfa.10235)
Supplement: Supplementary file 1 — Appendix S1. Supporting Information [file JSFA-100-2121-s001.doc]

| **Tab. S1a** | **S1** | **S2** | **S3** | **S4** | **S5** |
| --- | --- | --- | --- | --- | --- |
| **Copy number (*bar*)** | **5120** | **1280** | **320** | **80** | **20** |
| **RUN 1** | 26,75 | 28,65 | 30,70 | 32,67 | 34,07 |
|  | 26,90 | 28,87 | 30,82 | 32,84 | 34,42 |
|  | 26,81 | 28,66 | 30,89 | 32,66 | 34,25 |
|  | 26,93 | 28,89 | 30,81 | 32,90 | 34,89 |
|  | 26,89 | 28,79 | 30,83 | 32,63 | 35,39 |
|  | 26,93 | 28,81 | 30,80 | 32,67 | 34,89 |
| **RUN 2** | 26,77 | 28,57 | 30,77 | 32,64 | 35,34 |
|  | 26,85 | 28,84 | 30,77 | 32,85 | 34,59 |
|  | 26,79 | 28,84 | 30,87 | 32,85 | 35,57 |
|  | 26,90 | 28,86 | 30,66 | 32,84 | 35,07 |
|  | 26,84 | 28,84 | 30,86 | 32,70 | 34,44 |
|  | 26,93 | 28,83 | 30,80 | 32,74 | 34,82 |
| **RUN 3** | 26,64 | 28,59 | 30,60 | 32,54 | 35,13 |
|  | 26,94 | 28,91 | 30,76 | 32,71 | 35,20 |
|  | 26,84 | 28,81 | 30,82 | 32,73 | 34,77 |
|  | 26,83 | 28,89 | 30,78 | 32,51 | 35,19 |
|  | 26,84 | 28,86 | 30,82 | 32,84 | 35,97 |
|  | 26,98 | 28,99 | 30,79 | 32,99 | 35,05 |
| **RUN 4** | 26,67 | 28,72 | 30,68 | 32,72 | 35,05 |
|  | 26,89 | 28,91 | 30,58 | 33,17 | 34,59 |
|  | 26,82 | 28,84 | 30,85 | 33,01 | 34,52 |
|  | 26,75 | 28,92 | 30,73 | 32,69 | 34,98 |
|  | 26,83 | 28,83 | 30,96 | 32,85 | 35,37 |
|  | 26,86 | 28,92 | 30,78 | 32,67 | 34,59 |
|  |  |  |  |  |  |
| **Tab. S1b** | **Average Cq (1)** | **Average Cq (2)** | **Average Cq (3)** | **Average Cq (4)** | **Log copy n.** |
| **S1** | 26,87 | 26,85 | 26,84 | 26,80 | 3,709 |
| **S2** | 28,77 | 28,80 | 28,84 | 28,86 | 3,107 |
| **S3** | 30,81 | 30,79 | 30,76 | 30,77 | 2,505 |
| **S4** | 32,73 | 32,77 | 32,72 | 32,85 | 1,903 |
| **S5** | 34,65 | 34,97 | 35,22 | 34,85 | 1,301 |

| **Tab. S1c** | **S1** | **S2** | **S3** | **S4** | **S5** |
| --- | --- | --- | --- | --- | --- |
| **Copy number (*pat*)** | **5120** | **1280** | **320** | **80** | **20** |
| **RUN 1** | 26,71 | 28,65 | 30,64 | 32,69 | 34,42 |
|  | 26,66 | 28,64 | 30,75 | 32,34 | 35,49 |
|  | 26,59 | 28,61 | 30,69 | 32,70 | 35,18 |
|  | 26,60 | 28,71 | 30,71 | 32,75 | 35,16 |
|  | 26,67 | 28,64 | 30,64 | 32,62 | 34,70 |
|  | 26,66 | 28,68 | 30,89 | 32,62 | 34,80 |
| **RUN 2** | 26,66 | 28,84 | 30,57 | 32,64 | 34,91 |
|  | 26,66 | 28,74 | 30,56 | 32,61 | 34,59 |
|  | 26,60 | 28,67 | 30,60 | 32,80 | 34,74 |
|  | 26,64 | 28,66 | 30,71 | 32,75 | 34,77 |
|  | 26,70 | 28,60 | 30,68 | 32,61 | 34,91 |
|  | 26,62 | 28,60 | 30,57 | 32,66 | 35,14 |
| **RUN 3** | 26,62 | 28,67 | 30,86 | 32,89 | 35,53 |
|  | 26,66 | 28,64 | 30,93 | 32,69 | 34,77 |
|  | 26,70 | 28,53 | 30,70 | 32,92 | 34,96 |
|  | 26,59 | 28,81 | 30,70 | 32,31 | 34,32 |
|  | 26,64 | 28,68 | 30,59 | 32,70 | 34,79 |
|  | 26,67 | 28,67 | 30,65 | 32,90 | 34,98 |
| **RUN 4** | 26,65 | 28,73 | 30,83 | 33,05 | 34,94 |
|  | 26,73 | 28,70 | 30,68 | 32,95 | 35,21 |
|  | 26,66 | 28,68 | 30,76 | 33,03 | 34,77 |
|  | 26,65 | 28,74 | 30,71 | 32,58 | 34,36 |
|  | 26,67 | 28,62 | 30,69 | 32,62 | 34,90 |
|  | 26,65 | 28,73 | 30,62 | 32,82 | 35,57 |
|  |  |  |  |  |  |
|  |  |  |  |  |  |
| **Tab. S1d** | **Average Cq (1)** | **Average Cq (2)** | **Average Cq (3)** | **Average Cq (4)** | **Log copy n.** |
| **S1** | 26,65 | 26,65 | 26,65 | 26,67 | 3,709 |
| **S2** | 28,66 | 28,68 | 28,67 | 28,70 | 3,107 |
| **S3** | 30,72 | 30,61 | 30,74 | 30,71 | 2,505 |
| **S4** | 32,62 | 32,68 | 32,73 | 32,84 | 1,903 |
| **S5** | 34,96 | 34,84 | 34,89 | 34,96 | 1,301 |

**Supplementary table 1.** PCR efficiency and linearity evaluation. Cq values for each dilution point (S1-S5) from each run (RUN 1-4) for the *bar* system (Tab. S1a) and the *pat* system (Tab. S1c). Average Cq of each dilution point *vs* the Log copy number (HGE) for the *bar* system (Tab. S1b) and the *pat* system (Tab. S1d).

| Tab. S2a | *bar* (Cq) | | | *pat* (Cq) | | |
| --- | --- | --- | --- | --- | --- | --- |
| Volume | 25µl | 24µl | 26µl | 25µl | 24µl | 26µl |
| 1 | 32,20 | 31,61 | 32,16 | 32,05 | 31,89 | 32,22 |
| 2 | 32,17 | 31,98 | 32,47 | 32,31 | 31,97 | 32,30 |
| 3 | 31,82 | 32,14 | 32,08 | 31,99 | 32,32 | 32,09 |

| **Tab. S2b** | *bar* (Cq) | | *pat* (Cq) | |
| --- | --- | --- | --- | --- |
| Mastermix | UMM | QM | UMM | QM |
| 1 | 32,20 | 31,93 | 32,05 | 33,10 |
| 2 | 32,17 | 32,00 | 32,31 | 33,23 |
| 3 | 31,82 | 31,94 | 31,99 | 33,13 |

| Tab. S2c | *bar* (Cq) | | *pat* (Cq) | |
| --- | --- | --- | --- | --- |
| Instrument | 7900HT | QS7 | 7900HT | QS7 |
| 1 | 32,20 | 31,15 | 32,05 | 31,25 |
| 2 | 32,17 | 31,07 | 32,31 | 31,23 |
| 3 | 31,82 | 31,26 | 31,99 | 31,28 |

Supplementary table 2. Robustness evaluation of the *pat/bar* duplex real time PCR assay.

Tab. S2a: Cq values obtained by testing three different reaction volumes; Tab. S2b: Cq values obtained by testing two different master mixes; Tab. S2c: Cq values obtained by testing two different instruments.

UMM: Universal Master Mix (Thermo Fisher Scientific); QM: QuantiTect Multiplex PCR NoROX Mastermix (Qiagen); QS7: QuantStudio 7 (Thermo Fisher Scientific)

| Species | GM Event (%) | *bar* | | *pat* | |
| --- | --- | --- | --- | --- | --- |
|  |  | Expected | Verified by IZSAM | Expected | Verified by IZSAM |
| Cotton | T304-40 (10%) | P | + | A | - |
|  | GHB119 (1%) | P | + | A | - |
|  | MON15985 (100%) | A | - | A | - |
| Maize | Bt176-Bt11 (1%) | P | + | P | + |
|  | MON810 (10%) | A | - | A | - |
|  | DAS59122 (1%) | A | - | P | + |
|  | Bt176 (1%) | P | + | A | - |
|  | Bt11 (1%) | A | - | P | + |
| Oilseed rape | MON88302 (100%) | A | - | A | - |
|  | T45-RF1 (100%) | P | + | P | + |
|  | T45 (100%) | A | - | P | + |
|  | RF1 (100%) | P | + | A | - |
| Soybean | DAS68416-4 (10%) | A | - | P | + |
|  | DAS44406 (0,1%) | A | - | P | + |
|  | MON89788 (100%) | A | - | A | - |
|  | MON87705 (100%) | A | - | A | - |
| Potato | EH92-527-1 (100%) | A | - | A | - |
| Sugar beet | H7-1 (100%) | A | - | A | - |
| Rice | LL62 (100%) | P | + | A | - |
| Cotton/soybean | GHB119-DAS81419 (10%) | P | + | P | + |

**Supplementary table 3.** Method transferability was tested by verification of the *pat/bar* duplex assay specificity on twenty blind samples P: Presence; A: Absence.


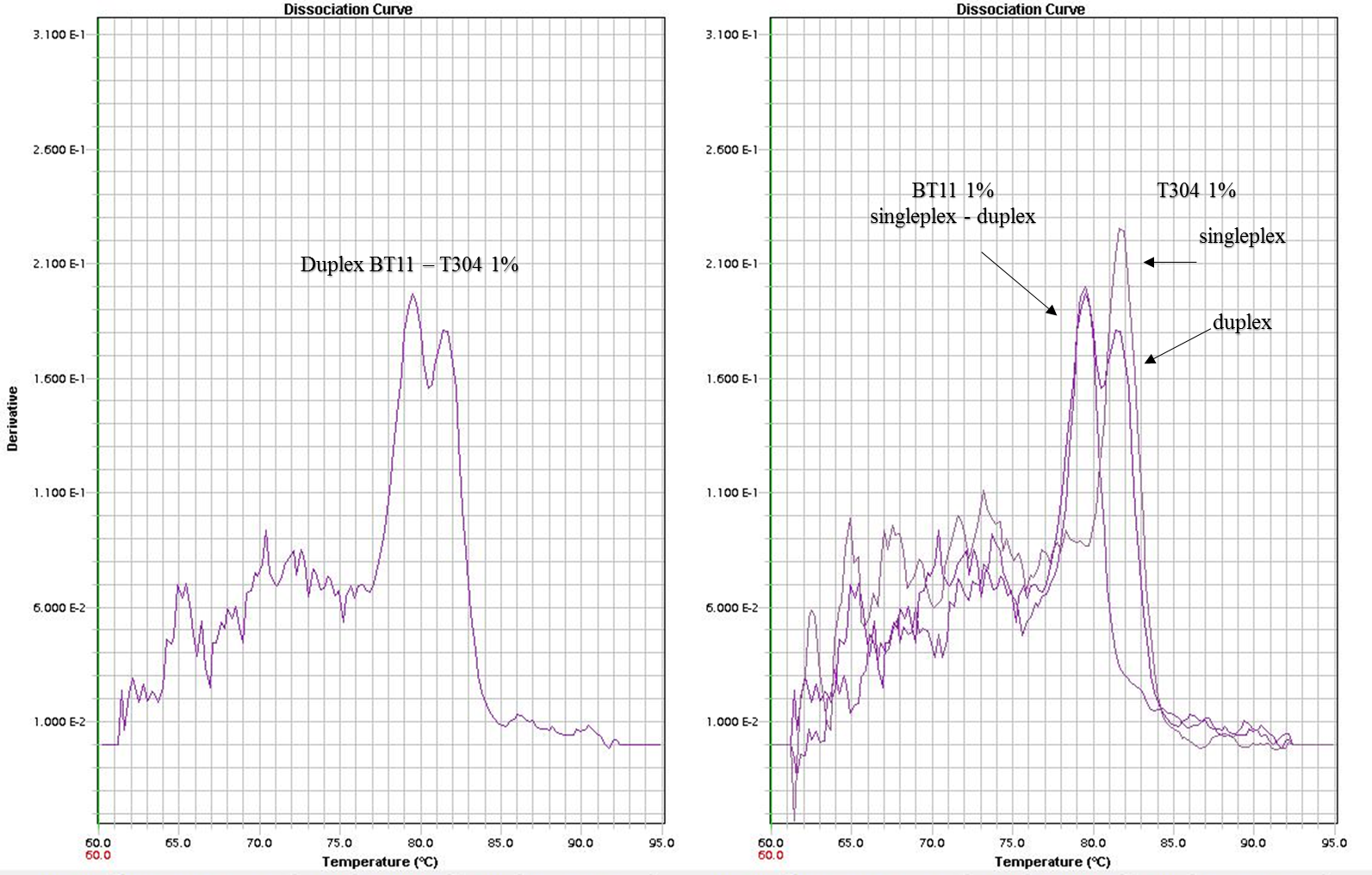


**Supplementary figure 1**: Dissociation curves after PCR amplification **a:** Duplex BT11 – T304 1% peaks**; b:** Comparison between duplex BT11 – T304 peaks and own singleplex peak.


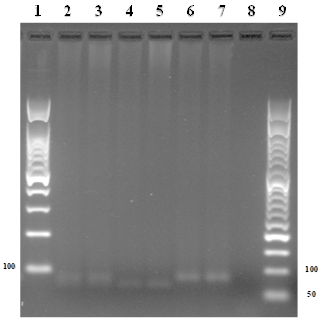


**Supplementary figure 2**: Specificity of the *pat/bar* duplex real time PCR assay.

The real time PCR products were separated by electrophoresis on a 2% agarose gel.

Lane 1: 100 bp DNA ladder; lane 2: BT176/BT11 1%; lane 3: T45/RF1 100%; lane 4: BT176 100%; lane 5: RF1 100%; lane 6: BT11 1%; lane 7: T45 100%; lane 8: NTC; lane 9: 50 bp DNA ladder.


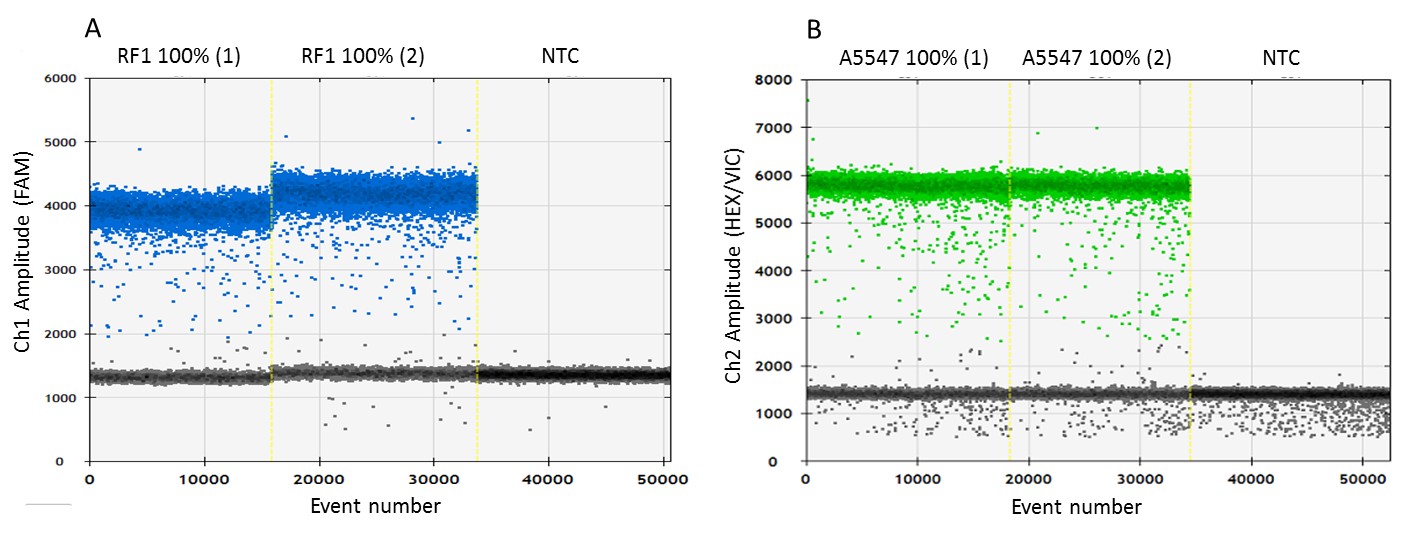


**Supplementary figure 3**: 1D-Amplitude plots of *bar* (A) and *pat* (B) ddPCR method applied to the certified reference materials (AOCS) from RF1 100% rapeseed and A5547 100% soybean (1:10 dilution of the extracted DNA). The figure shows the droplet populations (for single assay): blue and green dots represent FAM (channel 1) and YY (channel 2) positive droplets, respectively. Grey dots represent the negative ones.
